# A Multi-Scale Model for Predicting Physically Short Crack and Long Crack Behavior in Metals

**DOI:** 10.3390/ma17215163

**Published:** 2024-10-23

**Authors:** Xing Yang, Chunguo Zhang, Panpan Wu, Anye Xu, Pengfei Ju, Dandan Yang, Zhonghong Dong

**Affiliations:** 1 Key Laboratory of Highway Construction Technology and Equipment of the Ministry of Education, Chang’an University, Xi’an 710064, China; 2 China Construction First Group Corporation Limited, Xi’an 710075, China

**Keywords:** multi-scale model, physically short crack, material properties, transition criterion, Gaussian distribution theory

## Abstract

The fatigue behavior of metal specimens is influenced by defects, material properties, and loading. This study aims to establish a multi-scale fatigue crack growth model that describes physically short crack (PSC) and long crack (LC) behavior. The model allows the calculation of crack growth rates for uniaxial loading at different stress ratios based on the material properties and specimen geometry. Furthermore, the model integrates the Gaussian distribution theory to consider material heterogeneity and the experimental measurement errors that cause fatigue scatter. The crack growth rate and fatigue life of metal specimens with different notch geometry were predicted. The curves generated by the multi-scale model were mainly consistent with the test data from the published literature at the PSC and LC stages.

## 1. Introduction

Fatigue failure is the most common type of fracture in metallic materials and plays a crucial role in engineering applications. [1]. Statistical data indicates that up to 80% of failures during engineering component service life can be attributed to the initiation of fatigue short cracks (SC) [2]. Predicting the fatigue behavior of SC is of paramount importance due to the inevitable presence of SC effects in structures or components during service; maintenance intervals are predicated on the assessment of crack growth rates. Traditional damage tolerance approaches based on long cracks (LC) may lead to inaccuracies in the estimation of fatigue crack growth (FCG) rate or fatigue life. Therefore, from the perspective of structural integrity and safety, incorporating SC behavior into crack growth models is of paramount importance.

Numerous experimental studies indicate that SC fatigue propagation can be composed of two different stages: microstructurally short crack (MSC) and physically short crack (PSC) [3]. These detailed characteristics can be described as follows: (1) In the MSC stage, the crack initiates and propagates through the maximum shear stress plane, and the crack length has the same scale as the grain size [4]. The MSC initiation and propagation behavior are mainly affected by microstructural factors such as grain size [5], crystal orientation [6], and grain boundary (GB) [7]; (2) In the PSC stage, the crack propagates in a plane perpendicular to the direction of applied tensile stress; the crack length is longer than MSC but empirically less than 1mm, or ten times the grain size [8]. Due to the size range of certain initial damage defects (such as voids, inclusions, and scratches) falling within the PSC stage, the initiation point of material fatigue life is often marked in most cases. Some studies suggest that the PSC stage accounts for a considerable part of total fatigue life [9,10]. Thus, the ultimate goal is to establish a unified multi-scale FCG model within the framework of fracture mechanics to elucidate the PSC and LC behaviors during the fatigue damage process.

Tremendous efforts have been dedicated to developing models that elucidate the propagation of both SC and LC. Based on linear elastic fracture mechanics (LEFM), the long crack phase can be effectively described by Paris’ law [11], which was the first to introduce fracture mechanics into the description of fatigue crack growth. However, Paris’ law only provides a correlation between fatigue and experimental data and lacks predictive capability for materials, rendering it unsuitable for direct application to SC. On the other hand, due to the failure of the similitude concept, the notion of the stress intensity factor based on LEFM cannot be directly applied to the SC stage. Thus, numerous research studies have been conducted to investigate the propagation behavior of SC. Some researchers attempted to extend crack growth models to the SC stage by modifying the LC driving force or resistance. Chan and Lankford [12] propose the microstructure dissimilitude model, which deduces the SC driving force by describing the local yield strengths of the crack tip. Based on the Zheng–Hirt model, Chapetti [13,14] proposed a PSC propagation model that incorporates the concept of crack closure. Bang [15] utilized a dual-parameter model with Δ*K* and *K*_max_ to characterize the propagation behavior of SC and LC. Furthermore, several studies explored alternative driving parameters to replace the stress intensity factor (SIF), including the J-integral [16], strain energy density [17], and crack tip displacement [18].

In recent years, significant efforts have been made to investigate the multi-scale fatigue crack propagation behavior in metallic materials [19,20,21]. Notably, the propagation behavior of MSC is primarily influenced by microstructural features, while the propagation behavior of PSC/LC can be described using the LEFM theory [22,23]. Despite these advancements, current research still faces the following limitations: (i) Determining the transition criterion between SC and LC has seen limited theoretical investigation. This transition criterion, however, critically influences the accuracy of multi-scale model assessments of fatigue behavior; (ii) The material’s fatigue resistance performance is related to its fracture toughness; applied load, crack closure, and specimen geometry primarily influence the crack driving force. However, some experimental results [10,20] indicate that the FCG rate during the SC stage is also influenced by transition behavior and mechanical properties (e.g., yield strength). The comprehensive effects of these factors on FCG have not yet been thoroughly explained; (iii) For power-law forms of PSC/LC propagation models, there exist fitting parameters *C*, and *m*, based on experimental conditions, which lack a solid physical foundation. It is well known that fitting parameters can limit the application of the model. Therefore, a multi-scale analytical model is necessary.

In this study, the transition criterion between the PSC and LC stages was established by examining the FCG behavior of metal specimens. Building upon this, a unified multi-scale model for prediction of FCG rate during the total fatigue stages was formulated. This led to the proposal of a multi-scale FCG model influenced by strength, material characteristics, loading, and notch stress concentration factors. Additionally, based on the established multi-scale model, the physical meaning of parameters *C* and *m* has been discussed. Furthermore, considering the material heterogeneity and experimental measurement errors that contribute to FCG scatter, Gaussian distribution theory is incorporated with the multi-scale model. Additionally, based on experimental data for PSC and LC, predictions on crack growth rate and fatigue life under different stress ratios were provided.

## 2. Model Derivation

**Figure 1:**
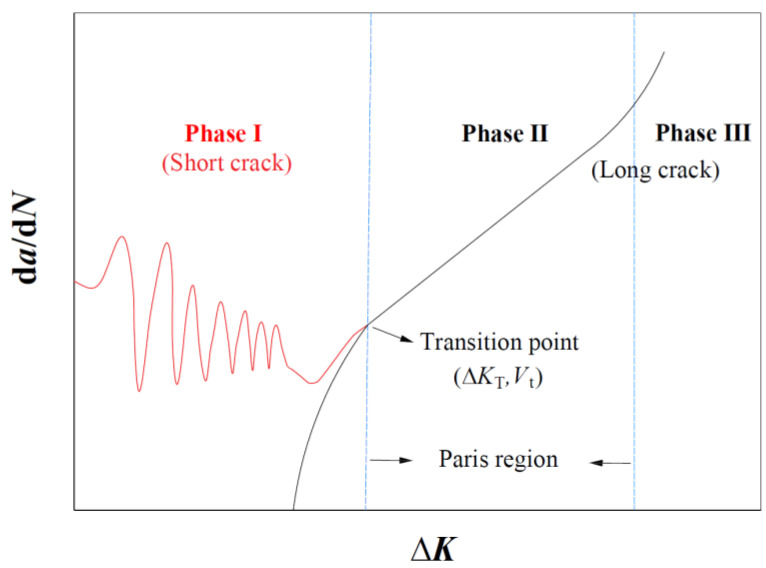
Fatigue crack growth curves of short crack and long crack.

As shown in Figure 1, Phase II can be depicted by Paris’ law. Considering the value of SIF threshold variation from the PSC stage to the LC stage under the influence of crack closure, the modified Paris–Erdogan equation is used to predict the FCG rate of Phase I and Phase II [23].

(1) $$\frac{\mathrm{da}}{\mathrm{dN}}=C\cdot {\left(\Delta K\cdot q-\Delta K_{\mathrm{th},\mathrm{SC}}\right)}^{m}$$

where *C* and *m* are experimental fitting parameters, ∆*K_th,sc_* is the SIF threshold of SC and LC stage [13], and *q* is the microstructural factor used to modify the driving force [12].

As illustrated in Figure 1, (∆*K*_T_, *V*_t_) serves as a transition point from Phase I to Phase II. ∆*K*_T_ can be calculated by considering the transition length *l* between the SC and LC stages.

(2) $$\Delta K_{T}=F(a)\cdot \Delta \sigma \cdot \sqrt{\pi l}$$

where F(*a*) is the notch geometry corrector [24], determined by notch geometry, *l* is calculated by following equations [25]:

(3) $$l=\frac{4\pi \cdot {\left(1+\nu \right)}^{2}\cdot D}{3}\cdot \left(\frac{h}{b}\right)\cdot {\left(\frac{\sigma_{\mathrm{eR}}}{\sigma_{a}}\right)}^{2}$$

where *b* is the Burgers vector, *ν* is Poisson’s ratio, the value of *b/h* ranges from 1.1547 for the fcc (facet-centered cubic) metals to 1.414 for bcc metals [26], *D* is equivalent grain size (the average grain size is usually used), *σ_eR_* is fatigue limit at stress ratio *R*, and *σ_a_* is applied stress amplitude (*σ_a_* = ∆*σ*/2).

By substituting (∆*K*_T_, *V*_t_) into Equation (1), the transition FCG rate *V*_t_ would be a certain value,

(4a) $$V_{t}=C\cdot {\left(\Delta K_{T}-\Delta K_{\mathrm{th},\mathrm{SC}}^{T}\right)}^{m}$$

Thus, parameter *C* can be calculated by:

(4b) $${C=V}_{t}/{\left(\Delta K_{T}-\Delta K_{\mathrm{th},\mathrm{SC}}^{T}\right)}^{m}$$

The aim in this paper is to find the physical meaning of parameters *C* and *m* and also explore the correlation between mechanical properties and the FCG rate. Based on Equation (4), we can obtain the following equation:

(5) $$\frac{\mathrm{da}}{\mathrm{dN}}{=V}_{t}\cdot {\left(\frac{\Delta K\cdot q-\Delta K_{\mathrm{th},\mathrm{SC}}}{\Delta K_{T}-\Delta K_{\mathrm{th},\mathrm{SC}}^{T}}\right)}^{m}$$

It should be mentioned that *V_t_* is a characteristic parameter in fatigue experiments. The fatigue life at the SC stage accounts for an important part of total fatigue stages; thus, the study on the transition zone between SC and LC is an important part of the complete problem in FCG. Based on the near-threshold model [27] and transition FCG model [28,29], the transition FCG rate *V*_t_ can be expressed as:

(6) $$V_{t}=\gamma \;\left(\sigma ,Y,M\right)$$

where *σ* is applied stress, such as ∆*σ* and *σ*_max_, *M* represents the material mechanical properties parameters, such as yield strength, tensile strength, fatigue strength, etc., and *Y* is a specimen parameter related to specimen geometry, such as notch type, notch depth, notch root radius, etc. Furthermore, in reference [28], the authors developed a model for the transitional crack growth rate between the second stage (stable propagation) and the third stage (rapid propagation) of long cracks. In the proposed model, the transitional crack growth rate is predominantly influenced by tensile strength and Young’s modulus; the transitional crack growth rate is accentuated with respect to the increasing in tensile strength; and the transitional crack growth rate is reduced with respect to the increasing in Young’s modulus. Thus, we hypothesize that the transition FCG rate *V*_t_ would be regarded as a characteristic parameter associated with material properties.

It is well known that the applied load and the notch geometry of the specimen significantly affect the FCG rate. Specifically, higher applied loads and sharper notches lead to increased crack growth rates. Additionally, Poisson’s ratio is positively correlated with the transverse strain perpendicular to the load. Therefore, in this study, the product of the maximum stress, notch stress concentration factor, and Poisson’s ratio is considered a positive correlating factor with the transition FCG rate. According to research in the literature [30], the yield strength and fatigue strength of materials respond to the localization of fatigue damage, and these two performance indicators also represent the material’s resistance to FCG. This implies that an increase in yield strength and tensile strength would reduce the transitional FCG rate. Consequently, based on the above discussion, and inspired by reference [28], it is reasonable to hypothesize that the transition crack growth rate can be expressed as follows:

(7) $$V_{t}=12\cdot V_{0}\cdot \exp\left(\frac{\nu \cdot {K_{t}}^{2}\cdot {\sigma_{\max}}^{2}}{\sigma_{y}\cdot \sigma_{\mathrm{eR}}}\right)$$

where V_0_ is the initial SC growth rate during ideal conditions (∆*K* = E√b [29], da/dn = *b*, and *b* is the Burgers vector), and *K*_t_ is the stress concentration factor of the specimen.

**Figure 2:**
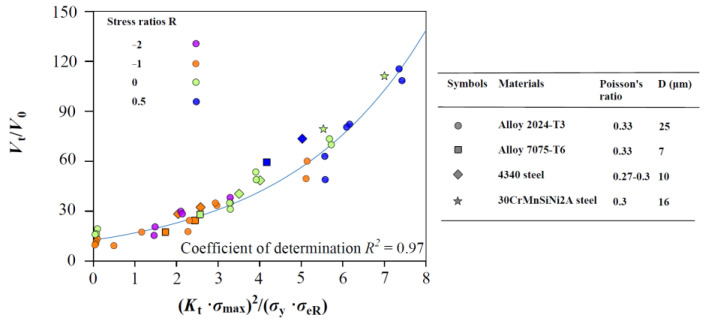
Predicted curves of Equation (7) at different stress ratios (color distinguished).

As schematically shown in Figure 2, FCG data of four kinds of materials (Al alloy 2024, Al alloy 7075, 4340 steel, and 30CrMnSiNi2A steel) at R from −2 to 0.5 were selected to compare the predicted curves [31,32,33,34,35,36]; the FCG data are distinguished by symbol and color, respectively. It is obvious that the test data are close to the predicted curve, and the coefficient of determination *R*^2^ = 0.97, which demonstrates the prediction of Equation (7) is in better agreement with the experimental data.

Therefore, combining Equations (2) and (7), the transition point (∆*K*_T_, *V*_t_) between SC and LC under d*a*/d*N*-∆*K* relation can be expressed by

(8) $$\left(F(a)\cdot \Delta \sigma_{\mathrm{eR}}\cdot \sqrt{\frac{{16\pi }^{2}\cdot {\left(1+\nu \right)}^{2}h\cdot d}{{3M}^{2}\cdot b}},12\cdot V_{0}\cdot \exp\left(\nu \cdot \frac{{K_{t}}^{2}\cdot {\sigma_{\max}}^{2}}{\sigma_{y}\cdot \sigma_{\mathrm{eR}}}\right)\;\right)$$

As discussed above, (∆*K*_T_, *V*_t_) would be a characteristic value related to loading, material properties, and specimen geometry. Thus, substituting Equation (7) into Equation (5), the multi-scale FCG rate model can be driven as below:

(9) $$\frac{\mathrm{da}}{\mathrm{dN}}{=V}_{t}\cdot {\left(\frac{\Delta K\cdot q-\Delta K_{\mathrm{th},\mathrm{SC}}}{\Delta K_{T}-\Delta K_{\mathrm{th},\mathrm{SC}}^{T}}\right)}^{m}=12\cdot b\cdot \exp\left(\nu \cdot \frac{{K_{t}}^{2}\cdot {\sigma_{\max}}^{2}}{\sigma_{y}\cdot \sigma_{\mathrm{eR}}}\right)\cdot {\left(\frac{\Delta K\cdot q-\Delta K_{\mathrm{th},\mathrm{SC}}}{\Delta K_{T}-\Delta K_{\mathrm{th},\mathrm{SC}}^{T}}\right)}^{m}$$

In most fatigue experimental studies, the fitted value of *m* varies from 1.4 to 4 [37]. To elucidate the relationship between *m* and material properties as well as experimental conditions, reference [38] is considered, which determines that the initiation FCG rate (∆*K* = *E*√*b*) is equal to the Burgers vector *b*; substituting (*E*√*b*, *b*) into Equation (9), the parameter *m* is expressed by:

(10) $$m=\ln\frac{b}{V_{t}}/\ln\frac{E\sqrt{b}\cdot q-\Delta K_{\mathrm{th},\mathrm{SC}}}{\Delta K_{T}-\Delta K_{\mathrm{th},\mathrm{SC}}^{T}}$$

## 3. Model Validation and Discussion

FCG data on notched specimens from four kinds of materials with *R* from −2 to 0.5 in the literature are digitized and used to validate Equation (9). The corresponding parameters of material properties are listed in Table 1, and the experiment details of loading and specimen geometry are listed in Table 2.

**Figure 3:**
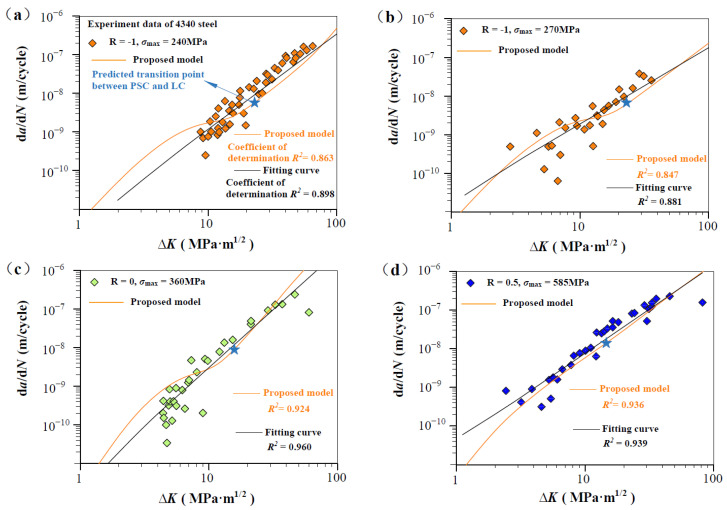
Prediction d*a*/d*N*-∆*K* curves at R = −1, 0, and 0.5 for 4340 steel specimens [36]: (**a**,**b**) predicted d*a*/d*N*-∆*K* curves at R = −1, *σ*_max_ = 240 MPa, and R = −1, *σ*_max_ = 270 MP; (**c**) predicted d*a*/d*N*-∆*K* curves at R = 0, *σ*_max_ = 360 MPa; and (**d**) predicted d*a*/d*N*-∆*K* curves at R = 0.5, *σ*_max_ = 585 MPa.

**Figure 4:**
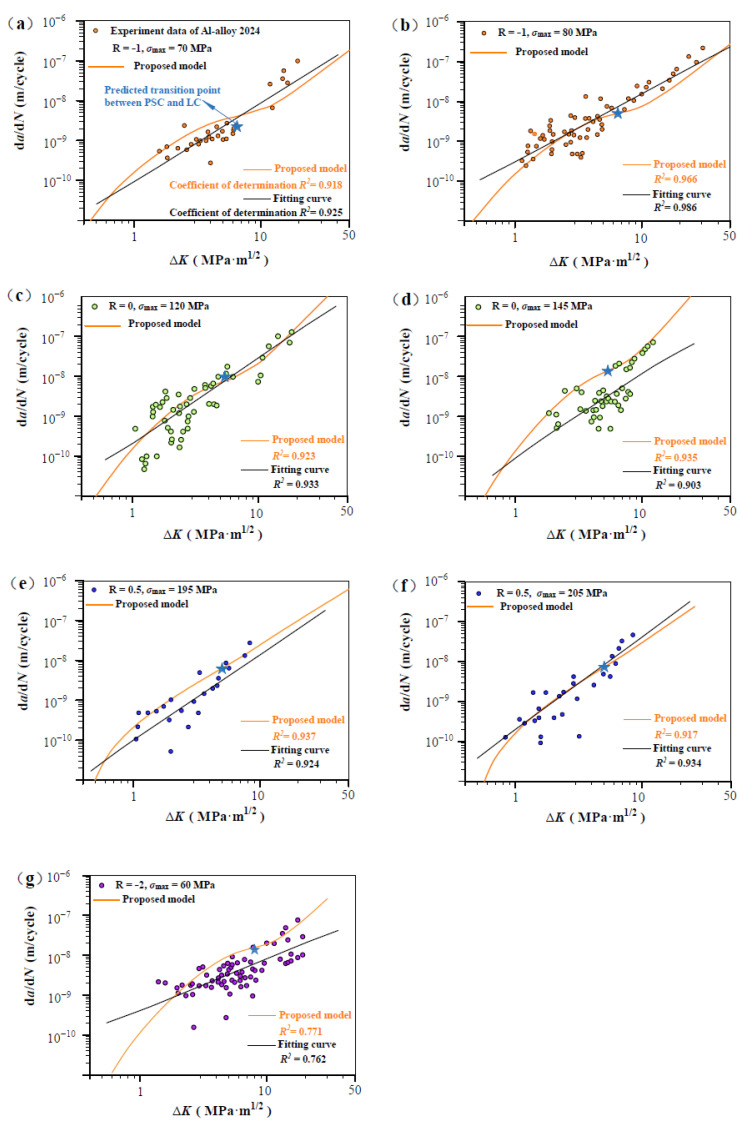
Prediction d*a*/d*N*-∆*K* curves at R = −1, 0, 0.5, and −2 for Al alloy 2024 specimens [32]: (**a**,**b**) predicted d*a*/d*N*-∆*K* curves at R = −1, *σ*_max_ = 70 MPa, and R = −1, *σ*_max_ = 80 MP; (**c**,**d**) predicted d*a*/d*N*-∆*K* curves at R = 0, *σ*_max_ = 120 MPa, and R = 0, *σ*_max_ = 145 MP; (**e**,**f**) predicted d*a*/d*N*-∆*K* curves at R = 0.5, *σ*_max_ = 195 MPa, and R = 0.5, *σ*_max_ = 205 MP; and (**g**) predicted d*a*/d*N*-∆*K* curves at R = −2, *σ*_max_ = 60MPa.

**Figure 5:**
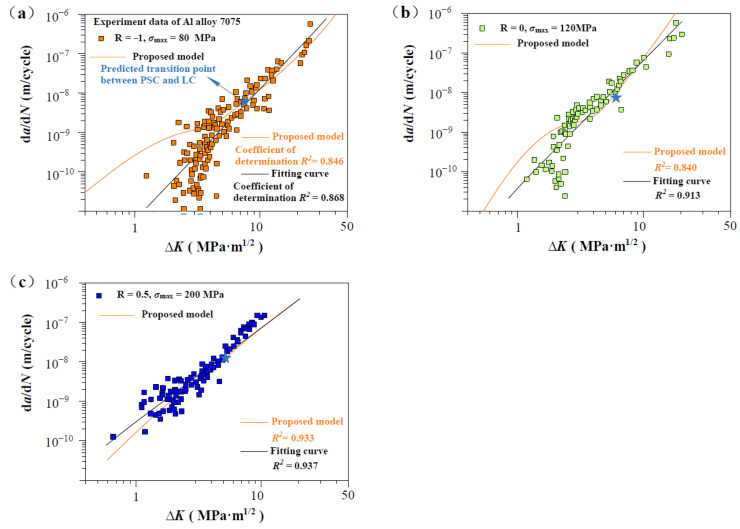
Prediction d*a*/d*N*-∆*K* curves at R = −1, 0, and 0.5 for Al alloy 7075 specimens [31]: (**a**) predicted d*a*/d*N*-∆*K* curves at R = −1, *σ*_max_ = 80 MP; (**b**) predicted d*a*/d*N*-∆*K* curves at R = 0, *σ*_max_ = 120 MPa; and (**c**) predicted d*a*/d*N*-∆*K* curves at R = 0.5, *σ*_max_ = 200 MP.

**Figure 6:**
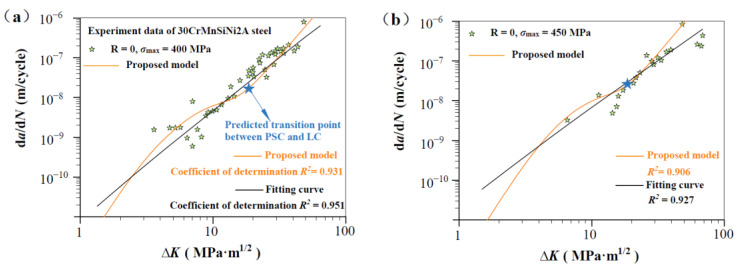
Prediction d*a*/d*N*-∆*K* curves at R = 0 for 30CrMnSiNi2A steel specimens [35]: (**a**,**b**) predicted d*a*/d*N*-∆*K* curves at R = 0, *σ*_max_ = 400 MPa, and R = 0, *σ*_max_ = 450 MP.

Figure 3, Figure 4, Figure 5 and Figure 6 show the predicted d*a*/d*N*-∆*K* curves using Equation (9) with different *R* together with experimental results. The predicted curves and FCG data are distinguished by symbol and color. It is obvious that the proposed model successfully predicts the FCG behavior from PSC to LC under different stress ratios. To validate the accuracy of the proposed model, we compared it with a power-law fitting curve (black line). As shown in Figure 3, Figure 4, Figure 5 and Figure 6, the correlation coefficient values for the predicted and fitted curves of four materials under different stress ratios were calculated. It is noteworthy that the fitted curves were derived using the power-law form of the Paris model, and the results indicate that the predicted curves (in orange) exhibit accuracy close to that of the fitted curves. The proposed model (orange line) demonstrated precision comparable to the power-law curve. The results indicate that Equation (12) can serve as a viable alternative to traditional fitting methods for accurately predicting the FCG behavior of metallic materials under different *R* values. Additionally, the predicted transition point (∆*K*_T_, *V*_t_) between PSC and LC is highlighted in Figure 3, Figure 4, Figure 5 and Figure 6. It can be seen that the fluctuation of the FCG data decreases after the transition point, which indicates that the scatter of test data differs significantly between the PSC and LC stages.

According to Equations (4b) and (10), C is analytically linked to transition conditions (V_t_, ∆K_T_, and ∆K^T^_th,sc_), and m is independent of stress ratio R and related to the Burgers vector, elasticity modulus, and threshold SIF range. In the calculations of this paper, most values of *m* fall between 1.8 and 3.5. For the same material, when the value of *m* is fixed, the variation in the value of *C* is primarily influenced by the transition crack growth rate.

Figure 3, Figure 4, Figure 5 and Figure 6 also show the fluctuation of crack propagation, which weakens with the crack length increase. Considering the influence of material heterogeneity and machining errors on experimental measurements of specimens, fatigue scatter is inevitable. Accordingly, to reconcile the discrepancy between the measured and theoretical FCG rate, a nondimensional parameter *β* is introduced by logarithmically transforming the Equation (9):

(11) $$\ln\frac{\mathrm{da}}{\mathrm{dN}}=\beta \cdot \ln\left\{V_{t}\cdot {\left(\frac{\Delta K\cdot q-\Delta K_{\mathrm{th},\mathrm{SC}}}{\Delta K_{T}-\Delta K_{\mathrm{th},\mathrm{SC}}^{T}}\right)}^{m}\right\}$$

**Figure 7:**
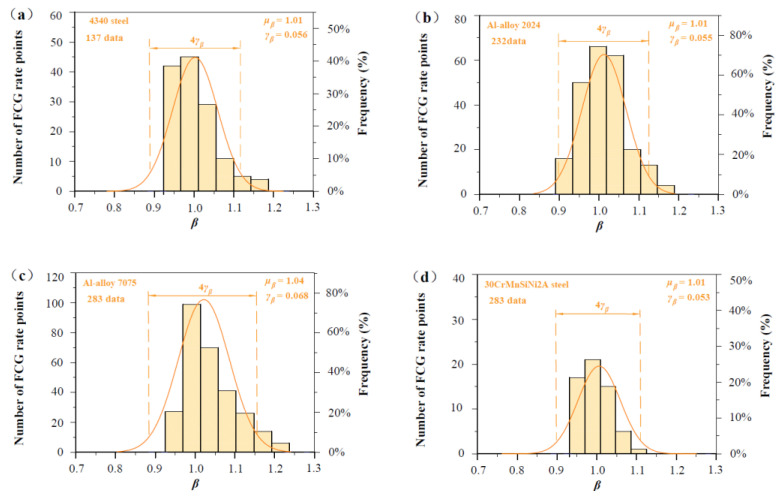
Histogram and normal distribution analysis of *β* values: (**a**) FCG data analysis of 4340 steel specimens; (**b**) FCG data analysis of Al alloy 2024 specimens; (**c**) FCG data analysis of Al alloy 7075, specimens. (**d**) FCG data analysis of 30CrMnSiNi2A steel specimens.

**Figure 8:**
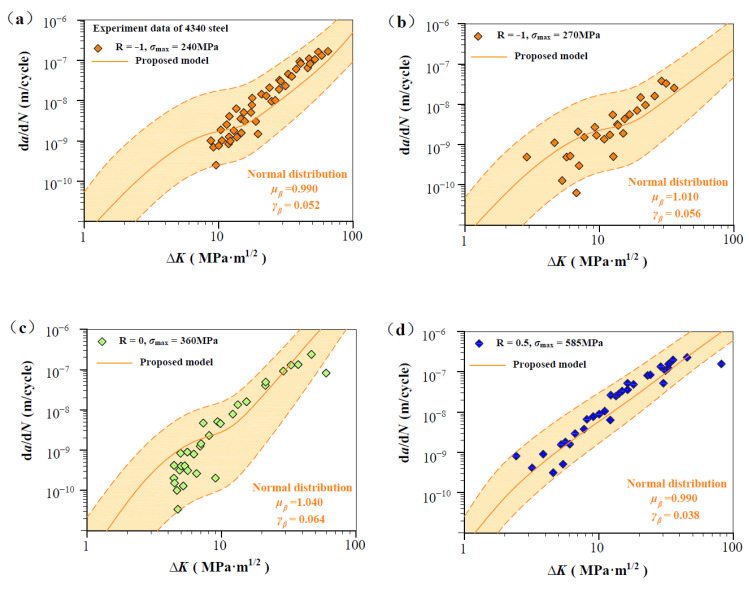
Normal distribution analysis of measurements on 4340 steel specimens. (**a**,**b**) Normal distribution analysis at R = −1, *σ*_max_ = 240 MPa, and R = −1, *σ*_max_ = 270 MP; (**c**) Normal distribution analysis at R = 0, *σ*_max_ = 360 MPa; and (**d**) Normal distribution analysis at R = 0.5, *σ*_max_ = 585 MPa.

**Figure 9:**
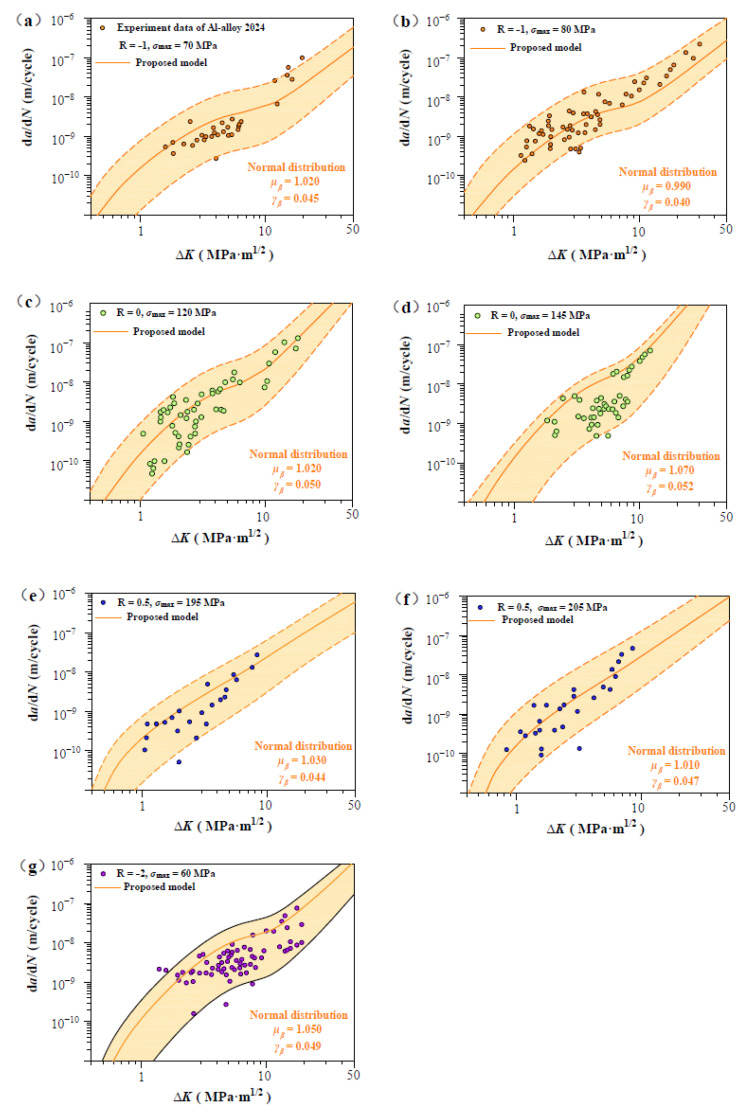
Normal distribution analysis of measurements on Al alloy 2024 specimens (**a**,**b**) Normal distribution analysis at R = −1, *σ*_max_ = 70 MPa, and R = −1, *σ*_max_ = 80 MP; (**c**,**d**) Normal distribution analysis at R = 0, *σ*_max_ = 120 MPa, and R = 0, *σ*_max_ = 145 MP; (**e**,**f**) Normal distribution analysis at R = 0.5, *σ*_max_ = 195 MPa, and R = 0.5, *σ*_max_ = 205 MP; and (**g**) Normal distribution analysis at R = −2, *σ*_max_ = 60 MPa.

**Figure 10:**
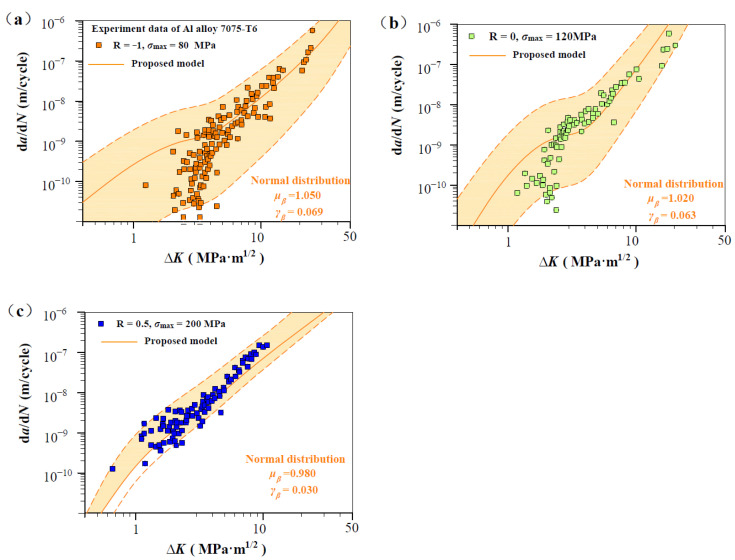
Normal distribution analysis of measurements on Al alloy 7075 specimens. (**a**) Normal distribution analysis at R = −1, *σ*_max_ = 80 MP; (**b**) Normal distribution analysis at R = 0, *σ*_max_ = 120 MPa; and (**c**) Normal distribution analysis at R = 0.5, *σ*_max_ = 200 MP.

**Figure 11:**
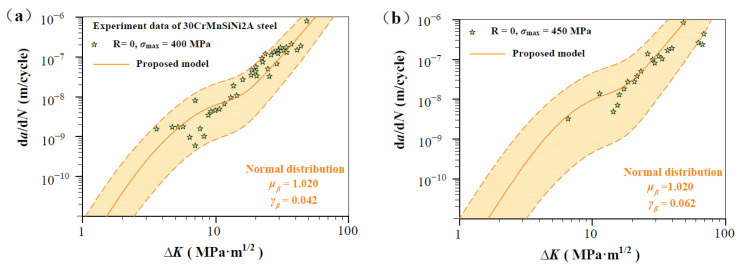
Normal distribution analysis of measurements on 30CrMnSiNi2A steel specimens. (**a**,**b**) Normal distribution analysis at R = 0, *σ*_max_ = 400 MPa, and R = 0, *σ*_max_ = 450 MP.

As shown in Figure 7, Figure 8, Figure 9, Figure 10 and Figure 11, four groups of d*a*/d*N*-∆*K* data are analyzed to show *β* fluctuation. Figure 7 shows the histogram and normal distribution analysis of four materials. Observations reveal that normal distribution curves align well with corresponding histograms, suggesting that the Gaussian distribution theory aptly describes the variability in experimental data. Subsequently, a probabilistic multi-scale model, achieving 96% reliability, is presented as follows:

(12) $$\frac{\mathrm{da}}{\mathrm{dN}}=\exp\left\{{(\mu }_{\beta }\pm {2\times \gamma }_{\beta })\cdot \ln\left[V_{t}\cdot {\left(\frac{\Delta K\cdot q-\Delta K_{\mathrm{th},\mathrm{SC}}}{\Delta K_{T}-\Delta K_{\mathrm{th},\mathrm{SC}}^{T}}\right)}^{m}\right]\right\}$$

where *μ*_β_ is the mean value of *β*, and *γ*_β_ is the standard deviation of *β*.

Figure 8 shows predicted d*a*/d*N*-∆*K* curves of AISI 4340 steel at stress ratios from −1 to 0.5 by Equation (12) using the transition crack growth rate *V*_t_. Similarly, predicted d*a*/d*N*-∆*K* curves are shown in Figure 9 for Al alloy 2024 at *R* from −1 to 0.5, Figure 10 for Al alloy 7075 at *R* from −1 to 0.5, and Figure 11 for 30CrMnSiNi2A steel at *R* = 0. As shown in Figure 8, Figure 9, Figure 10 and Figure 11, the scattered FCG data are mostly covered in the envelope area between the upper and lower boundaries. This implies that the nondimensional parameter *β* can effectively reflect the scatter of crack growth.

In Section 2, ∆*K*_T_ is calculated by transition crack length *l*. Considering the maximum stress, fatigue limit, yield strength, and notch geometry influences, the explicit equation related to transition FCG rate *V*_t_ is proposed, as shown in Equation (7). Based on this, parameter *C* is linked to the transition FCG rate *V*_t_, and *m* is determined by (∆*K*_T_, *V*_t_,) and (∆*K*_T_, *V*_t_,). In this study, we utilized the semi-empirical FCG model [23] as a framework, validating the universality of the theoretical parameters *C* and *m* using 17 groups of FCG data across four metals with *R* from −2 to 0.5. The error bands predicted by Equation (12) can cover the majority of the experimental data. Consequently, this model serves as a unified framework for predicting the FCG behavior of different metallic materials.

According to the above discussion, the proposed model eliminates the need for curve fitting, allowing for direct estimation of FCG behavior by using relatively accessible material property parameters, obviating the need for fatigue experiments. This advancement offers insights into the fatigue design of critical aerospace structures such as aircraft wings and fuselage structures. Furthermore, its predictions regarding the transition FCG rate provide valuable references for design and maintenance.

## 4. Conclusions

This study presents a multi-scale model for predicting fatigue cracks for four kinds of materials, incorporating both PSC and LC. The main conclusions are as follows:

(1) The proposed multi-scale model avoids the curve fittings of different stress ratios in the PSC and LC stages, exhibiting predictive capabilities comparable to the fitting curves.
(2) In the multi-scale model, parameter *C* is predominantly influenced by the transition crack growth rate *V*_t_, and *m* is independent of stress ratio *R*, and related to the Burgers vector, elasticity modulus, and threshold SIF range.
(3) By integrating the theory of normal distribution with Equation (9), a probabilistic model with 96% reliability for assessing the FCG rate was developed, which reflects the inevitability of fatigue scatter.

## Figures and Tables

**Table 1:** Material characteristic parameters were used in this study.

Materials	Grain Size (μm)	Burgers Vector *b* (m)	Poisson’s Ratio	Yield Strength (MPa)
Al alloy 2024 [32]	25	2.86 × 10^−10^	0.33	355
Al alloy 7075 [31]	7	2.86 × 10^−10^	0.33	520
AISI 4340 Steel [36]	16	2.48 × 10^−10^	0.27–0.3	1413
30CrMnSiNi2A steel [35]	10	2.52 × 10^−10^	0.3	1189

**Table 2:** The experimental parameters used in this study.

Materials	*R*	Load Type	*σ*_max_ (MPa)	*a*_0_ (mm)	*ρ* (mm)	*K*_t_	*σ_eR_* (MPa)	Notch Geometry
AISI4340 [36]	−1	AX	240	3.18	3.18	3.3	218	SENT
			270	3.18	3.18	3.3		
	0	AX	360	3.18	3.18	3.3	285	
	0.5	AX	585	3.18	3.18	3.3	526	
Al alloy 2024 [32]	−1	AX	70	3.18	3.18	3.17	62.5	SENT
			80	3.18	3.18	3.17		
	0	AX	120	3.18	3.18	3.17	104	
			145	3.18	3.18	3.17		
	0.5	AX	195	3.18	3.18	3.17	193	
			205	3.18	3.18	3.17		
	−2	AX	60	3.18	3.18	3.17	48.4	
Al alloy 7075 [31]	−1	AX	80	3.2	3.2	3.15	70.4	SENT
	0	AX	120	3.2	3.2	3.15	107	
	0.5	AX	200	3.2	3.2	3.15	183	
30CrMnSiNi2A steel [35]	0	AX	400	3.2	3.2	3.3	265	SENT
			450	3.2	3.2	3.3		

## Data Availability

The data presented in this study are available in a publicly accessible repository. The original data presented in the study are openly available in [NASA Technical Reports Server] at [Search—NASA Technical Reports Server (NTRS)].

## Nomenclature

*b*	Burgers vector
*ν*	Poisson’s ratio
*h*	Slip band width
*l*	Transition length between PSC and LC
*a*	Crack length
*a*_0_	Notch depth
*ρ*	Notch root radius
*β*	Nondimensional parameter
*μ*_β_	Mean value of β
*γ*_β_	Standard deviation of β
*σ*_a_	Stress amplitude
*σ*_max_	Maximum stress
*σ*_eR_	Plain fatigue limit at stress ratio R
*Y*	Geometry factor
*R*	Stress ratio
*E*	Elasticity modulus
*D*	Grain size
Δ*σ*	Stress range
Δ*K*	Stress intensity factor range
∆*K*_T_	Stress intensity factor range for physically short crack to long crack
∆*K_th,sc_*	Fatigue threshold stress intensity factor range for short crack
∆K^T^*_th,sc_*	Fatigue threshold stress intensity factor range for physically short crack to long crack

## Abbreviations

MSC	Microstructurally short crack
PSC	Physically short crack
SC	Short crack
LC	Long crack
FCG	Fatigue crack growth
LEFM	Linear elastic fracture mechanics
SIF	Stress intensity factor
AX	Axial loading
SENT	Single edge notch tension
